# Editorial: Enhancing therapeutic strategies: a focus on pharmacovigilance in new wave antidepressants and antipsychotics

**DOI:** 10.3389/fpsyt.2025.1706980

**Published:** 2025-11-18

**Authors:** Octavian Vasiliu, Mirko Manchia, Yoshihiro Noguchi, Georgios Mikellides

**Affiliations:** 1 Clinical Neurosciences Department, Carol Davila” University of Medicine and Pharmacy, Bucharest, Romania; 2 Department of Psychiatry, Dr. Carol Davila” University Emergency Central Military Hospital, Bucharest, Romania; 3 Section of Psychiatry, Department of Public Health and Medical Services, University of Cagliari, Cagliari, Italy; 4 Department of Pharmacology, Dalhousie University, Halifax, NS, Canada; 5 Gifu Pharmaceutical University, Gifu, Japan; 6 Department of Basic and Clinical Sciences, Medical School, University of Nicosia, Nicosia, Cyprus; 7 Centre for Repetitive Transcranial Magnetic Stimulation, Cyprus rTMS, Nicosia, Cyprus

**Keywords:** zuranolone, BDNF, atypical antipsychotics, drug interactions, escitalopram, vortioxetine, pharmacovigilance, cariprazine

## 1 Introduction

Psychopharmacology has undergone transformative advancements, particularly with the development of new wave antidepressants and antipsychotics. Such progress was required, and still is, due to the challenges in the clinical domain, where nearly one-third of patients living with severe mental disorders (SMDs) experience some degree of resistance to treatment (1–3). The limited efficacy of antipsychotic treatment may be detected from the first episode of psychosis (FEP), with meta-analytical research reporting pooled rates of remission of 58% and pooled prevalence of recovery of no more than 38% (3). When referring to Major Depressive Disorders (MDDs), the Sequenced Treatment Alternatives to Relieve Depression (STAR*D) trial highlighted that only 33% of the patients recovered after the first trial of antidepressants, and many patients required multiple trials, with the rates of responsiveness decreasing as the number of trials increased (4, 5).

The consequences of these limitations on treatment efficacy when schizophrenia spectrum disorders (SSDs) and MDDs are approached are reported on multiple, relevant outcomes, such as quality of life, daily functionality and disability rates, frequency of hospitalization and relapse, risk of complications, and mortality rates (6–8). Based on the negative consequences of treatment resistance in SMDs, significant efforts are being made to develop new pharmacological agents that could address the unmet need for better interventions, starting with the very first episode of a mental disorder. However, advances in the field of psychopharmacology should not be understood as the creation of other “me too” drugs that can only perpetuate the illusion of therapeutic solutions; rather, they should involve the exploration of new pathogenic models for SMD (9–11).

Over the past decade, new-wave antidepressants, i.e., esketamine, zuranolone, brexanolone, toludesvenlafaxine, gepirone, and the dextromethorphan plus bupropion combo, and new-wave antipsychotics, such as lumateperone, cariprazine, blonanserin, and pimavanserin, have been approved by drug regulatory authorities (e.g., the U.S. Food and Drug Administration - FDA, the European Medicines Agency - EMA, the Japanese Pharmaceuticals and Medical Devices Agency- PMDA, and the Chinese National Medical Products Administration - NMPA) (12–14). Some of these innovative therapies are characterized by novel mechanisms of action, such as glutamatergic modulation in the case of esketamine or antagonism of serotonin 5HT2A and 2C receptors without significant action on dopamine D2 receptors in the case of pimavanserin, and offer new hope for addressing the persistent challenges of depressive and psychotic disorders. Beyond novel antidepressants and antipsychotics, psychedelics are being revisited as promising alternatives for treatment-resistant depression (15), heralding yet another class of agents for which post-approval monitoring of safety, identity effects, and adverse events will be essential.

**Figure 1:**
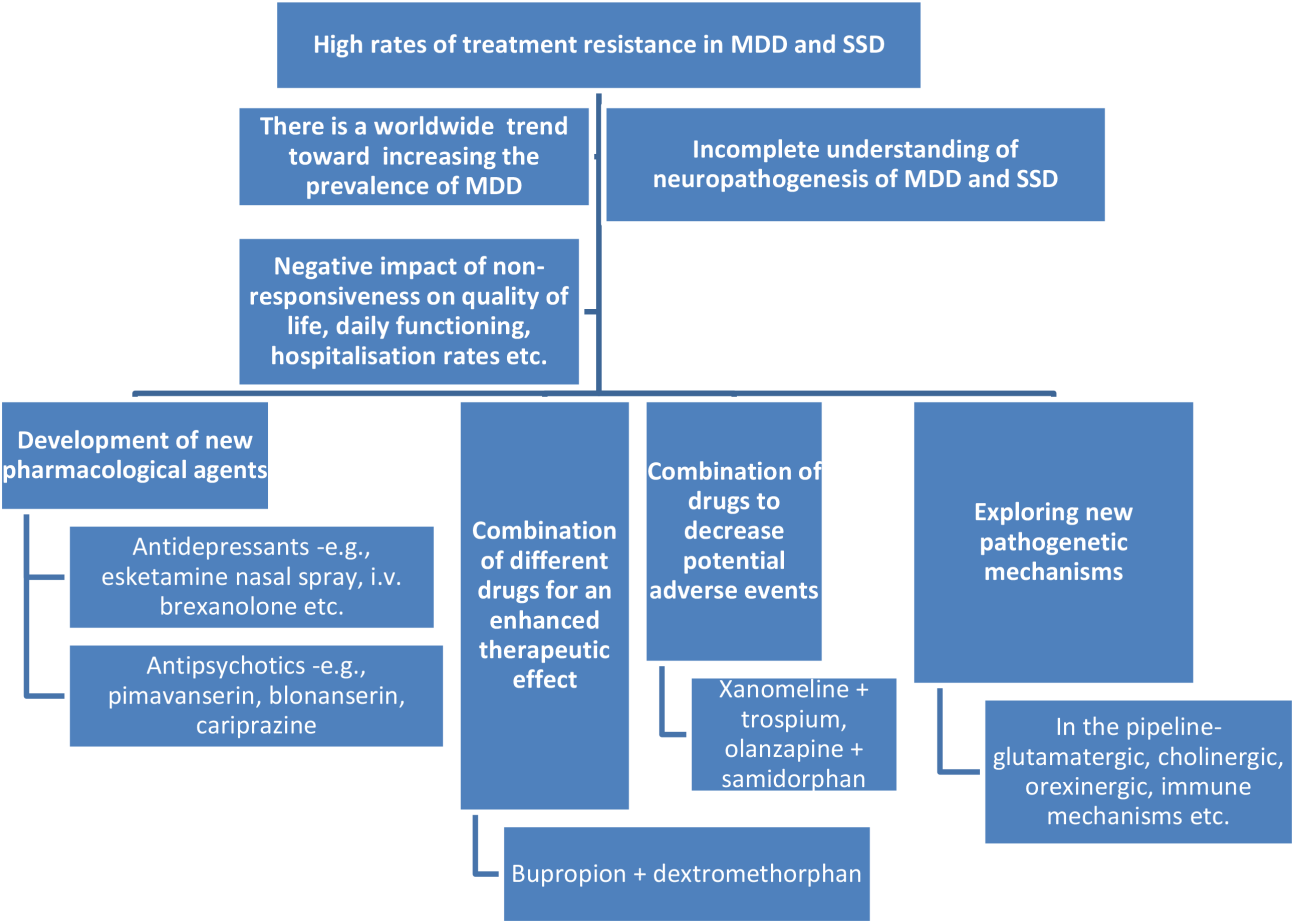
The need to develop new wave psychotropic drugs and pharmacovigilance studies. MDDs, major depressive disorders; SSDs, schizophrenia spectrum disorders.

Despite this progress, significant gaps regarding the real-life efficacy and tolerability of these agents remain; therefore, the purpose of this Research Topic was to gather new data on the pharmacovigilance studies regarding the new wave of antidepressants and antipsychotics (**Figure 1**).

## 2 Emerging areas of research in the field of eating disorders as a public health challenge

Pejušković et al. presented, in a well-structured manner, the motives for using cariprazine as a treatment for various depression symptom clusters, which is a topic with significant clinical implications given the partial or complete lack of responsiveness to currently available antidepressants. Cariprazine is a new-wave antipsychotic with selective action on dopamine D3 receptors, a mechanism that deserves more attention for its potential as an add-on strategy for antidepressant therapy. The authors showed that cariprazine could be particularly beneficial for patients with MDD exhibiting anhedonia, cognitive deficits, and possibly fatigue and hypersomnia. Also, evaluating cariprazine’s efficacy across these symptom domains could reveal patterns that support more personalized treatment approaches for depression.

Zeng et al. published a study on the antidepressant effects and inflammatory profile of Sini San, a traditional Chinese medicine formula composed of Radix Bupleuri, Paeoniae Radix Alba, Aurantii Fructus Immaturus, and *Glycyrrhizae Radix et Rhizoma* (licorice). Their study examined the effects of Sini San on chronic unpredictable mild stress (CUMS) in rats, highlighting the need to find translational models of MDD. Sini San was able to modulate the gut-brain axis by inhibiting the NLPR3 inflammasome, thus decreasing CUMS-induced inflammation and gut microbiota dysbiosis in this preclinical model. This effect could be useful due to the potential significance of regulating the Brain-Derived Neurotrophic Factor (BDNF)/Tropomyosin receptor kinase B(TrkB)/Phosphatidylinositol 3-kinase (PI3K)/Protein kinase B (AKT) signaling pathway in treating depressive symptoms.

Huang et al. explored the postmarketing safety of zuranolone, a neuroactive steroid that acts as a positive allosteric modulator of the gamma-aminobutyric acid (GABA) A receptors, by searching the FDA Adverse Event Reporting System (FAERS) database from the third quarter of 2023 to the second quarter of 2024. Based on an analysis of 154 reports primarily suspecting zuranolone and 426 adverse events from a total of 1,626,204 adverse event (AE) reports, zuranolone administration was found to be associated mainly with AEs in the domain of “Nervous system disorders and Psychiatric disorders”. Specific AEs included in this report were somnolence, dizziness, fatigue, sedation, suicidal ideation, tremor, abnormal feelings, headache, anxiety, and nausea.

Alsfouk et al. conducted a cross-sectional study (N = 220 patients) to investigate the pharmacokinetic and pharmacodynamic interactions between antipsychotics and concomitant drugs using electronic Lexicomp^®^, and assessed their risk rating, severity, and reliability. Antidepressants (20%), anticonvulsants (18%), and cardiovascular agents (15%) were the most frequently administered concomitant drugs, and the rate of potential drug-drug interactions was 71%. There was a “fair” level of scientific evidence in 64% of cases and “good” in 36% of interactions. The most frequent potential adverse effects were increased sedation (36%), hyperglycemia (15%), and decreased blood pressure (14%). This study convincingly illustrates the importance of understanding the potential pharmacologic interactions between antipsychotics and concomitant drugs, and of considering the use of AI technology to assist in this endeavor.

The retrospective study by Cavanah et al. focused on patterns in (es)citalopram, a selective serotonin reuptake inhibitor, prescriptions for Medicaid and Medicare patients in the United States (between 2015 and 2020) through the perspective of the “evergreening” effect, a phenomenon that refers to a strategy by the pharmaceutical companies of extending their drug’s patent by making minor modifications to the original product, formulation, or delivery method. A decreasing tendency for citalopram and an increasing one for escitalopram prescription rates were noted in both medical insurance databases during the researched interval. There were reported differences between generic and brand products for both substances, with generic forms being less expensive than the brand-name versions. The authors showed that the noticeable decline in the use of citalopram that co-occurred with an increase in escitalopram prescriptions supports the “evergreening” hypothesis.

Zhang et al. authored a pharmacovigilance study that consisted of collecting data from FAERS, starting in the third quarter of 2013 up to the first quarter of 2024, regarding the relevant AEs associated with vortioxetine, a multimodal serotonergic antidepressant. A total of 11,298 cases were analyzed as “primary suspected” for vortioxetine hydrobromide, with AEs for this agent involving 27 systemic organ classes. A total of 150 significantly disproportionate preferred terms met all four predefined algorithms.

Furutani and Nagoshi showed in their case report that two patients diagnosed with somatic symptom disorder could benefit from treatment with vortioxetine, regardless of their age (one patient was 88 years old, and the other was 29 years old) or specific clinical manifestations. The rapid effect of this multimodal serotonergic antidepressant was accompanied by good tolerance, highlighting its efficacy, especially in patients who had an insufficient response to serotonin reuptake inhibitors.

## 3 Conclusions

This Research Topic emphasizes the need for pharmacovigilance studies in the case of new wave antidepressants and antipsychotics that have appeared in clinical use in the last decade. Large databases such as FAERS are extremely useful for researchers investigating newly reported AEs, and based on the synthesis of these reports, periodic revisions to these products’ summaries of characteristics are granted (Zhang et al., Huang et al.). Also, the need to investigate new pathogenetic hypotheses for MDDs and SSDs is a constant challenge for researchers (Zeng et al.) who want to develop new, innovative therapeutic agents while avoiding pitfalls such as the “me too” phenomenon, or “evergreening” (Cavanah et al.). New indications for the drugs such as vortioxetine for somatic symptom disorder or cariprazine as an add-on for MDD are certainly worthy of further investigation (Furutani and Nagoshi Y, Pejušković et al.). Also, a thorough investigation of drug-drug interactions when co-prescribing antipsychotics and other pharmacological agents is a clinical necessity if AEs, early discontinuation, lack of efficacy, or toxicity are to be avoided (Alsfouk et al.).

The articles published in this Research Topic suggest that pharmacovigilance studies in the fields of MDDs and SSDs are strongly needed to fully understand their clinical and pharmacological profiles beyond the pivotal trials required by drug regulatory agencies.
